# The TIRS trial: protocol for a cluster randomized controlled trial assessing the efficacy of preventive targeted indoor residual spraying to reduce *Aedes*-borne viral illnesses in Merida, Mexico

**DOI:** 10.1186/s13063-020-04780-7

**Published:** 2020-10-08

**Authors:** Pablo Manrique-Saide, Natalie E. Dean, M. Elizabeth Halloran, Ira M. Longini, Matthew H. Collins, Lance A. Waller, Hector Gomez-Dantes, Audrey Lenhart, Thomas J. Hladish, Azael Che-Mendoza, Oscar D. Kirstein, Yamila Romer, Fabian Correa-Morales, Jorge Palacio-Vargas, Rosa Mendez-Vales, Pilar Granja Pérez, Norma Pavia-Ruz, Guadalupe Ayora-Talavera, Gonzalo M. Vazquez-Prokopec

**Affiliations:** 1 Unidad Colaborativa de Bioensayos Entomológicos, Campus de Ciencias Biológicas y Agropecuarias, Universidad Autónoma de Yucatán, Merida, Mexico; 2 Department of Biostatistics, University of Florida, Gainesville, FL 32611 USA; 3 Center for Inference and Dynamics of Infectious Diseases, Seattle, WA 98109 USA; 4 Vaccine and Infectious Disease Division, Fred Hutchinson Cancer Research Center, Seattle, WA 98109 USA; 5 Department of Biostatistics, University of Washington, Seattle, WA 98109 USA; 6 Emerging Pathogens Institute, University of Florida, Gainesville, FL 32611 USA; 7 Hope Clinic of the Emory Vaccine Center, Division of Infectious Diseases, Department of Medicine, School of Medicine, Emory University, Decatur, GA 30030 USA; 8 Department of Biostatistics and Bioinformatics, Rollins School of Public Health, Emory University, Atlanta, GA 30322 USA; 9 Health Systems Research Center, National Institute of Public Health, Cuernavaca, Mexico; 10 Centers for Disease Control and Prevention, Atlanta, GA USA; 11 Department of Biology, University of Florida, Gainesville, FL 32611 USA; 12 Department of Environmental Sciences, Math and Science Center, Emory University, 400 Dowman Drive, 5th floor, Suite E530, Atlanta, GA 30322 USA; 13 Centro Nacional de Programas Preventivos y Control de Enfermedades (CENAPRECE) Secretaría de Salud Mexico, Mexico City, Mexico; 14 Secretaria de Salud de Yucatan, Merida, Yucatan Mexico; 15 Centro de Investigaciones Regionales Hideyo Noguchi, Universidad Autonoma de Yucatan, Merida, Mexico

**Keywords:** Cluster randomized, Insecticide, *Aedes aegypti*, Dengue, Chikungunya, Zika, Indoor, Urban

## Abstract

**Background:**

Current urban vector control strategies have failed to contain dengue epidemics and to prevent the global expansion of *Aedes*-borne viruses (ABVs: dengue, chikungunya, Zika). Part of the challenge in sustaining effective ABV control emerges from the paucity of evidence regarding the epidemiological impact of any *Aedes* control method. A strategy for which there is limited epidemiological evidence is targeted indoor residual spraying (TIRS). TIRS is a modification of classic malaria indoor residual spraying that accounts for *Aedes aegypti* resting behavior by applying residual insecticides on exposed lower sections of walls (< 1.5 m), under furniture, and on dark surfaces.

**Methods/design:**

We are pursuing a two-arm, parallel, unblinded, cluster randomized controlled trial to quantify the overall efficacy of TIRS in reducing the burden of laboratory-confirmed ABV clinical disease (primary endpoint). The trial will be conducted in the city of Merida, Yucatan State, Mexico (population ~ 1million), where we will prospectively follow 4600 children aged 2–15 years at enrollment, distributed in 50 clusters of 5 × 5 city blocks each. Clusters will be randomly allocated (*n* = 25 per arm) using covariate-constrained randomization. A “fried egg” design will be followed, in which all blocks of the 5 × 5 cluster receive the intervention, but all sampling to evaluate the epidemiological and entomological endpoints will occur in the “yolk,” the center 3 × 3 city blocks of each cluster. TIRS will be implemented as a preventive application (~ 1–2 months prior to the beginning of the ABV season). Active monitoring for symptomatic ABV illness will occur through weekly household visits and enhanced surveillance. Annual sero-surveys will be performed after each transmission season and entomological evaluations of *Ae. aegypti* indoor abundance and ABV infection rates monthly during the period of active surveillance. Epidemiological and entomological evaluation will continue for up to three transmission seasons.

**Discussion:**

The findings from this study will provide robust epidemiological evidence of the efficacy of TIRS in reducing ABV illness and infection. If efficacious, TIRS could drive a paradigm shift in *Aedes* control by considering *Ae. aegypti* behavior to guide residual insecticide applications and changing deployment to preemptive control (rather than in response to symptomatic cases), two major enhancements to existing practice.

**Trial registration:**

ClinicalTrials.gov NCT04343521. Registered on 13 April 2020. The protocol also complies with the WHO International Clinical Trials Registry Platform (ICTRP) (Additional file 1).

**Primary sponsor:**

National Institutes of Health, National Institute of Allergy and Infectious Diseases (NIH/NIAID).

## Background

*Aedes*-borne viruses (ABVs; e.g., dengue [DENV], chikungunya [CHIKV], Zika [ZIKV]) pose a major public health burden worldwide [1–3]. Transmitted primarily by the highly anthropophilic mosquito *Aedes aegypti*, ABVs propagate epidemically, inflicting substantial healthcare and development costs on urban tropical populations. Model projections estimate that an average of 390 million DENV infections occur per year, of which 96 million manifest clinically [4, 5]. Explosive DENV outbreaks saturate healthcare systems [6], with worldwide estimates as high as $39 billion (2010 US$) per year spent on costs related to medical care, surveillance, vector control, and lost productivity [7]. The emergence and rapid epidemic propagation of CHIKV and ZIKV (and particularly congenital Zika) have added significant burden and costs to healthcare systems [8, 9]. Given the heavy global burden of ABV illness, and in the absence of efficacious vaccines or other therapeutic options, implementation of highly effective and currently available vector control strategies represents the most viable approach for ABV prevention [10, 11].

Vector control methods such as larval control, source reduction, and space spraying are widely used against ABVs [12, 13]. Unfortunately, there is limited epidemiological evidence that these methods are adequate to prevent or reduce human ABV transmission in a sustainable manner [13, 14]. Poorly designed evaluations, a historical lack of focus on quantifying intervention impact using epidemiological endpoints, and limited funding for large-scale randomized controlled trials with epidemiological endpoints have all contributed to the lack of rigorous, evidence-based, assessments of ABV vector control interventions [10, 15]. Furthermore, the classic deployment of house-based interventions in response to reported clinical ABV cases has failed to account for the important contribution of out-of-home human exposure to *Ae. aegypti* [16] and the silent contribution of asymptomatic infections in sustaining infectious virus in local mosquitoes [17]. Novel vector control approaches and intervention delivery strategies with proven and robust epidemiological evidence of their impact on ABV transmission are urgently needed.

Indoor residual spraying (IRS) is the use of long-lasting residual insecticides applied to the walls, eaves, and ceilings of houses or structures targeting vectors that land or rest on these surfaces [18–20]. The residual component of the application means that, for several weeks or months, the insecticide will kill mosquitoes and other insects that come into contact with treated surfaces. Historical evidence has shown that, when expeditiously implemented, residual insecticide applications can significantly reduce ABV transmission [21–23]. Despite this evidence, the fact that it is time consuming and dependent on specialized human resources has limited widespread adoption of IRS by ABV control programs due to the perceived challenge of scaling-up the intervention over large urban areas.

**Fig. 1:**
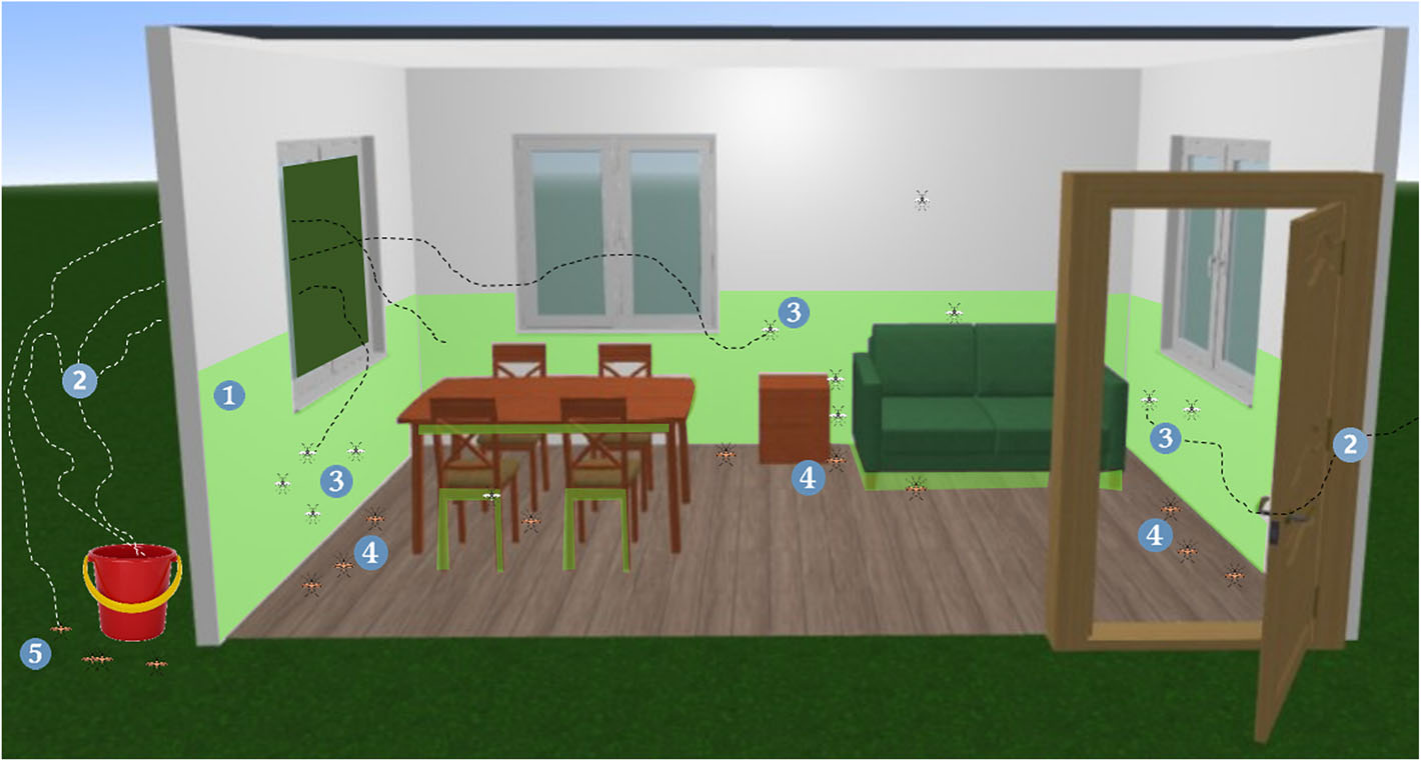
Targeted indoor residual spraying (TIRS) to control *Ae. aegypti*. In urban environments, houses are primarily built of brick and cement, and *Ae. aegypti* rests preferentially below 1.5 m of height. Spraying residual insecticides in walls below 1.5 m and in key resting sites such as under furniture (#1 in figure, represented in green) will eventually kill *Ae. aegypti* that may be emerging from immature larval habitats outdoors (2) and rest indoors on treated surfaces (3). After exposure to the residual insecticide, mortality can occur immediately (4) or after several hours/days (5)

In urban settings, adult *Ae. aegypti* typically rest indoors, where they feed frequently and almost exclusively on human blood [24]. Studies performed in Panama, Peru, and Mexico have shown that *Ae. aegypti* rest predominantly below heights of 1.5 m, mainly inside bedrooms and on surfaces made of cement, wood, and cloth [25–27]. Selectively applying residual insecticides below 1.5 m and on common mosquito resting surfaces provides an entomological impact similar to spraying entire walls (as performed in classic IRS), but in a fraction of the time (< 18%) and insecticide volume (< 30%) compared to classic IRS [28]. This selective insecticide application mode is called “targeted indoor residual spraying” (TIRS), and it involves the application of residual insecticides on exposed lower sections of walls [< 1.5 m], under furniture, and on dark surfaces throughout houses with the exception of the kitchen (Fig. 1). As such, TIRS is a rational vector control approach whereby *Ae. aegypti* resting behavior guides targeted insecticide applications, thus reducing unnecessary exposure to chemicals for both applicators and household residents (Fig. 1), and also reducing the time it takes to spray a premise with no apparent loss in insecticidal efficacy [28].

In Cairns, Australia, an observational study found that TIRS can reduce the probability of future DENV transmission by 86–96% as compared to unsprayed premises [29]. Concurrent trap collections of *Ae. aegypti* in the heart of the outbreak showed that TIRS was associated with a ~ 70% reduction in gravid *Ae. aegypti* female abundance [30]. In Merida, Mexico, a Phase II cluster randomized controlled trial (CRCT) evaluated the entomological impact of IRS with bendiocarb (Ficam®, Bayer, a carbamate insecticide to which local *Ae. aegypti* are fully susceptible) and reported reductions in indoor adult *Ae. aegypti* abundance up to 70% over a 3-month period, compared to no reduction when the pyrethroid deltamethrin was used [31]. Fitting such entomological information to an agent-based model of Yucatan State, Mexico, showed that high levels of TIRS coverage (75% of houses treated once per year) applied preemptively before the typical dengue season (before July) could reduce DENV infections by 89.7% in year 1 and 78.2% cumulatively over the first 5 years of an annual program [32]. Such findings were confirmed with another modeling study comparing TIRS with indoor space spraying in Iquitos, Peru [33]. These findings suggest that preemptive TIRS may provide high short-term and long-term effectiveness in preventing ABVs in endemic areas where transmission is seasonal.

A systematic review has identified TIRS as a highly promising approach for ABV prevention [34], but highlighted the limited evidence for TIRS due to the absence of impact estimates from randomized controlled trials with epidemiological endpoints performed in endemic settings. The study protocol presented here introduces the design for a CRCT to test whether TIRS, applied preventively, reduces laboratory-confirmed cases of ABV illness and infection in the city of Merida, Yucatan State, Mexico. Trial endpoints are listed in Table 1 and the approaches followed to quantify them will be described in subsequent sections.

**Table 1:** Outcome measures for the trial

Endpoint	Name	Population	Brief description
Primary	Laboratory-confirmed *Aedes*-borne disease	2–15-year-olds at enrollment	Laboratory-confirmed (virologically [RT-PCR testing of acute samples] or serologically [IgM and IgG ELISA testing of paired acute and convalescent samples]) symptomatic DENV, CHIKV, or ZIKV
Secondary	Laboratory-confirmed *Aedes*-borne infection	2–15-year-olds at enrollment	Laboratory-confirmed (serologically, [IgG ELISA and neutralization testing of annual surveillance samples]) DENV, CHIKV, or ZIKV infection. A FRNT50 for one DENV serotype ≥ 4-fold the FRNT50 to the other 3 serotypes is considered DENV mono-immune seroconversion
Secondary	*Aedes aegypti* infection with *Aedes*-borne viruses	Female *Ae. aegypti* collected in central 3 × 3 blocks of each cluster	*Ae. aegypti* mosquito infection rates with DENV, CHIKV, and ZIKV (assessed by RT-PCR) from 10% of households
Secondary	*Aedes aegypti* infestation	*Ae. aegypti* collected in central 3 × 3 blocks of each cluster	*Ae. aegypti* indoor entomological indices (adult presence and abundance, female presence and abundance, blood-fed female and abundance) from 10% of households
Secondary	Community acceptability of TIRS	Head of household in clusters receiving TIRS	Households receiving the intervention will be asked about their response and issues with TIRS. Conducted on same houses where entomology occurs.
Secondary	Community impact of TIRS	All ages	Number of symptomatic ABV cases reported to the passive surveillance system, including children and adults, distributed in treatment and control clusters
Secondary	Safety profile	All houses in 5 × 5 block treatment clusters	Percentage of households receiving the intervention that had evidence of a reaction to the insecticide (assessed and confirmed by study doctors). All sprayed households are eligible.

## Methods/design

### Study area

Merida, the capital city of Yucatan State, is the largest urban center in the region with 892,000 inhabitants [35]. The city has a tropical climate characterized by a mean annual temperature of 25.9 °C and an annual precipitation of 1050 mm. Merida is endemic for ABVs, with DENV being persistently transmitted since 1979 and, more recently, co-circulating with CHIKV (since 2015) and ZIKV (since 2016) [36, 37]. ABV transmission in Merida is seasonal, beginning in July and peaking in October–November. Baseline serological information (captured by ELISA methods) on natural ABV infection rates has been collected from Merida in 2015–2016 through a school-based cohort that followed all family members living in the same household as the enrolled children [37–39]. In 2015, DENV seroprevalence in the cohort was 70.2%, which increased with age from 31% in 0–8-year-olds to 79% in adults ≥ 20 years. In 2015–2016, the incidence of lab-confirmed ABV illness in the cohort was 14.6 per 1000 person-years (95% CI 10.8, 19.2) [37]. The incidence of symptomatic dengue infections observed during the same period was 3.5 cases per 1000 person-years (95% CI 1.9, 5.9). The majority of seroconversions occurred in the younger age groups (≤ 14 years old) [37–39]. The incidence of symptomatic chikungunya illness was 8.6 per 1000 person-years (95% CI 5.8, 12.3) and the incidence rate of symptomatic Zika illness was 2.3 per 1000 person-years (95% CI 0.9, 4.5) [37]. Zika virus symptomatic attack rate in pregnant women from the cohort was 31% [40].

Data from ~ 40,000 geocoded DENV, 2273 ZIKV and 1101 CHIKV symptomatic cases captured by Mexico’s national passive surveillance system from 2008 to 2016 identified DENV transmission “hot-spots” in Merida (areas with higher-than-average numbers of cases), which overlapped with CHIKV and ZIKV hot-spots [36]. Combining these data with information from the cohort, we found that DENV seroprevalence rates are ~ 2× higher in hot-spot areas compared to other areas [36].

Merida also has entomological laboratory infrastructure and trained personnel to conduct and evaluate TIRS [28, 31]. The Collaborative Unit for Entomological Bioassays (UCBE) is a reference laboratory within the Autonomous University of Yucatan (UADY) and is currently a World Health Organization Good Laboratory Practice (GLP) site for evaluating insecticide products for vector control [41].

### Trial design

**Fig. 2:**
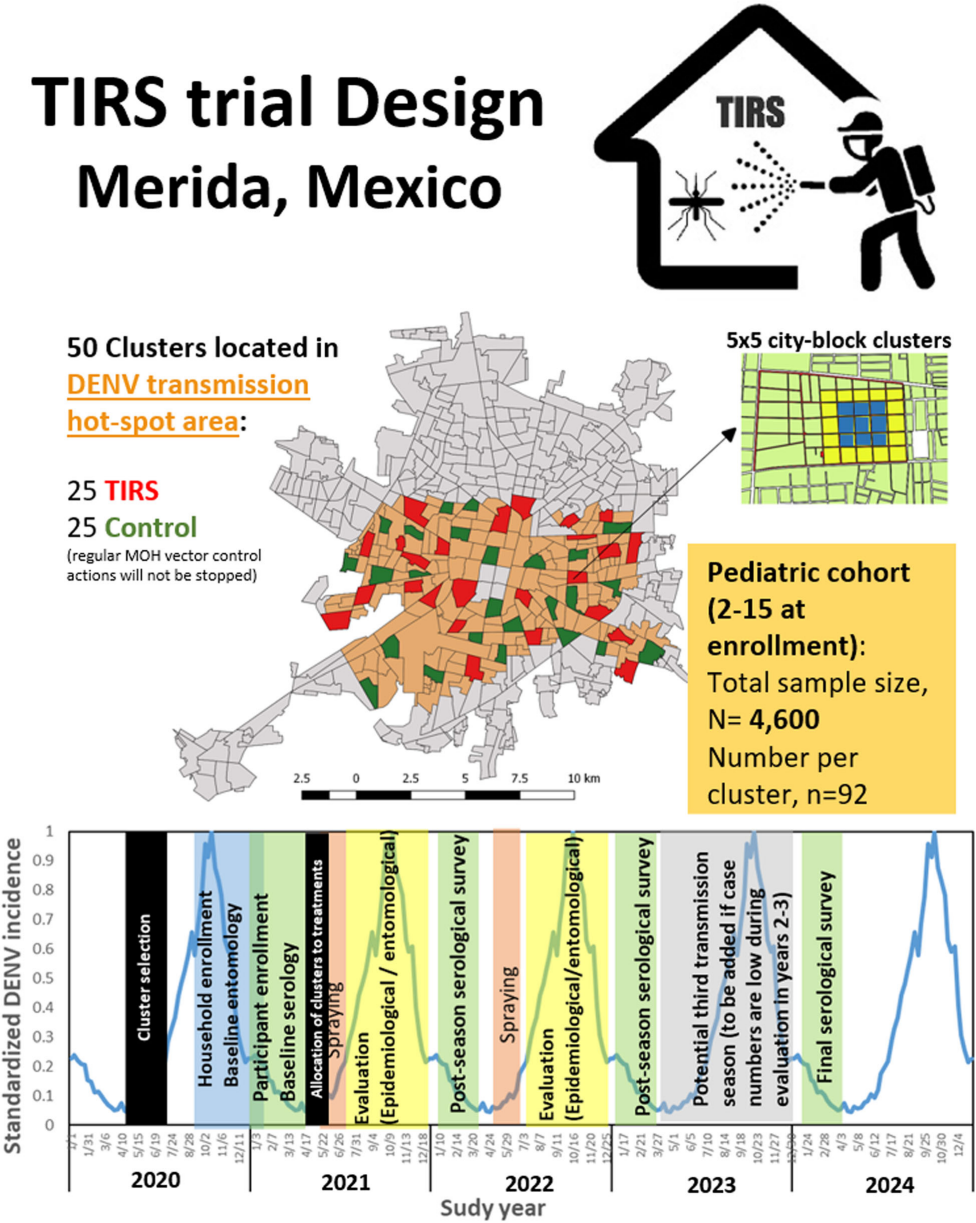
Proposed design for the TIRS trial. A possible arrangement of clusters within Merida, obtained using variable-constrained randomization. The final arrangement will be generated prior to household enrollment. Inset shows city blocks in yellow (the extent of the 5 × 5 cluster) with blue blocks showing the area where epidemiological and entomological evaluations will be concentrated (“fried egg” design). Blue lines in the lower panel show the 30-year average DENV case distribution by month, repeated on each trial year

The two-arm CRCT will include a total of 50 clusters of 5 × 5 city blocks each, with 25 clusters randomly allocated to the intervention (TIRS) arm and 25 clusters allocated to the control arm (Fig. 2). Routine Ministry of Health (MOH) vector control actions performed in response to symptomatic ABV cases reported to the healthcare system will not be interrupted and could occur across both study arms. Upon detection of a suspected ABV case in the national epidemiological database, Yucatan MOH mobilizes its staff aiming at containing local transmission by focusing efforts on adult mosquito control. Truck-mounted ULV spraying with the organophosphate insecticides chlorpyrifos and malathion is widely implemented in Merida, despite scientific evidence of its poor efficacy [34]. MOH response also involves indoor space spraying (ISS) with pyrethroids (mainly deltamethrin) and organophosphates (malathion) in houses that allow entry. Limitations in personnel, geographic extent of outbreaks, and availability of resources (e.g., insecticides) commonly challenge MOH operations, reducing the coverage and effectiveness of their actions [34]. All MOH actions will be mapped and included in secondary analyses evaluating the impact of TIRS in addition to routine vector control. Participants in both arms will have access to any concomitant care they may choose to pursue, including cleaning their own yard and eliminating mosquito breeding habitats or using commercially available insecticide sprays or repellents (e.g., transfluthrin coils).

Clusters will be located within the areas previously identified as hot-spots of ABV transmission [36] (Fig. 2). Placing all clusters within areas of high ABV incidence will increase power because of higher event rates and decrease the potential for imbalance across trial arms. To reduce contamination and edge effects, while all households in TIRS clusters will be offered the intervention, epidemiological and entomological evaluations will occur in the center of each cluster, following a “fried egg” design (Fig. 2). Entomological interventions that are constrained to a given area suffer from immigration of mosquitoes from untreated neighboring areas, as observed in a recent study that released *Wolbachia*-infected mosquitoes in Fresno, CA, and quantified mosquito dispersal up to 200 m from their release point [42]. By focusing participant enrollment on the central 3 × 3 blocks of the 5 × 5 clusters, we will minimize any contamination in our primary and secondary endpoints emerging from mosquitoes flying into treatment areas (Fig. 2). This “fried egg” design is novel for vector-borne diseases and has been proposed as a rational approach to quantify the epidemiological impact of vector control [10]. To prevent selection bias, enrollment into the trial will occur in all clusters before TIRS allocation has been determined.

### Power and sample size

To assess power and sample size requirements, we analyzed historical passive surveillance data from the 192 hot-spot census tracts with population size of at least 1000 (from our previous work characterizing the ABV hot-spot area [36]). We used yearly data from 2008 to 2016 on the number of dengue, chikungunya, and Zika cases recorded in children 0–14 years each year by census tract [36]. Data were combined into pairs of adjacent years to mimic a 2-year trial period, and Table 2 summarizes the mean incidence (number of cases over 2-year period/number of children) and intracluster correlation coefficient (ICC) for a given 2-year period [43]. Assuming 4% incidence over a 2-year period, 70% TIRS efficacy, an ICC of 0.035, and 20% loss to follow-up, we will require 92 age-eligible children enrolled per cluster for an overall sample size of 50 clusters and 4600 children to have 80% power to detect a significant reduction in ABV incidence between arms (Table 3).

**Table 2:** Data from 192 Merida census tracts located in the hot-spot area for ABV infection, showing the mean ABV incidence in 2-year pairs as well as the intracluster correlation coefficient (ICC)

Data source	Mean incidence	ICC
2008 + 2009 Dengue	0.0402	0.0345
2009 + 2010 Dengue	0.0530	0.0289
2010 + 2011 Dengue	0.0572	0.0164
2011 + 2012 Dengue	0.0847	0.0153
2012 + 2013 Dengue	0.0729	0.0188
2013 + 2014 Dengue	0.0455	0.0256
2014 + 2015 Dengue/Chik	0.0581	0.0229
2015 + 2016 Any	0.0385	0.0151

**Table 3:** Power calculations assuming 50 clusters allocated in a 1:1 ratio between treatment and controls to achieve power of 80%

TIRS efficacy	No. events	Total effective sample size	No. per cluster, unadjusted	No. per cluster, adjusted*	Total sample size, adjusted*
70%	28	1038	74	92	4600
75%	22	870	43	54	2700
80%	18	734	30	37	1850
90%	12	534	17	21	1050

*Adjusted for a 20% loss to follow-up

### Randomized allocation of the intervention

Clusters will be selected from the set of 190 census tracts within the ABV hot-spot area [36] that have a total population size of at least 1000 and at least 300 children aged 0–14 years, per the 2010 census (Fig. 2). Clusters are also selected to maximize the distance between the centroid of each cluster to the centroid of its nearest neighbor also in the trial. Given a set of 50 clusters, covariate-constrained randomization [44] will be used to limit imbalance across trial arms with respect to the following census tract-level variables: population size, per 2010 census; population density, per 2010 census; percent employed population, per 2010 census; and cumulative number of ABV cases between 2008 and 2016, per passive surveillance. These variables were selected because of their association with ABV transmission risk. For each balancing factor, only allocation patterns where the mean value of clusters in group A divided by the mean value of clusters in group B is within 1/1.1 to 1.1 are retained. Furthermore, we eliminate any allocation pattern with imbalance in the number of clusters per arm per sector greater than ± 1. To ensure randomization is not overly constrained, we only consider sets of 50 clusters that have many acceptable allocations into two groups of 25, satisfying validity criteria proposed by Moulton [44] (e.g., pairs of clusters always or never appearing in the same arm). Given the set of allocation patterns that meet the above balancing criteria, the biostatistics team at UF will use equal probability sampling to randomly select one allocation. A sample allocation pattern is plotted in Fig. 2. For participant enrollment, the study teams will be provided with a list of 50 census tracts for inclusion in the study, without a record of which census tracts are in group A or B. A random number generator produced by biostatisticians from UF will assign one group to TIRS and one group to control.

### Study participants

The trial will focus on the pediatric population, enrolling children aged 2–15 years in a longitudinal cohort to track their ABV illness and lab-confirmed seroconversion over two (and potentially three) transmission seasons (Fig. 2). The previously conducted cohort study in Merida indicated that the majority of dengue-naïve infections and seroconversions occurred in children ≤ 14 years old [37–39]. By following children aged 2–15 years at enrollment, we will capture the segment of the population with the highest probability of ABV illness. We excluded younger children (< 2 years) because of the difficulties in obtaining blood specimens and potential for cross-reactivity with maternal antibodies [45].

There will be two levels of participation: at the household level and at the individual child level. Table 4 shows the inclusion/exclusion criteria for each level. For each participation level, consent (and assent) will be obtained, as follows. On August 2020, after being given time to review information about the intervention, one adult household decision-maker will be asked for written consent to have their house included in the trial (at the time of consent, neither study personnel nor householders will know to which arm of the trial the house will be allocated). In consenting houses with children meeting the inclusion criteria (Table 4), individual consent/assent will be obtained during December 2020–January 2021. Parental informed consent will be obtained for children aged 2–10 years, and both assent to participate from children and a parental informed consent will be obtained for 11–15-year-olds (Additional file 2). Enrollment of children will be focused in the central 3 × 3 city blocks of each cluster and will extend beyond if not enough children are enrolled in the core. Consent will be obtained in participants’ homes. Study explanations will be provided to small groups of adults present in the household, whereas written consent and assent will be obtained from each individual participant.

**Table 4:** Inclusion and exclusion criteria for study enrollment

Inclusion criteria	Exclusion criteria
*Household level*	
Household is located within the bounds of a study cluster (5 × 5 city block clusters)	Households where study personnel identify a security risk (i.e., site where drugs are sold, residents are always drunk or hostile)
House located in a city block that has at least 60% residential premises	Sites where no residents spend time during the day (i.e., work 7 days a week outside the home)
	Inability for a resident to provide informed consent
	Non-residential places (e.g., businesses, schools, markets)
*Individual level*	
Aged 2 and up to 15 years at the time of initial enrollment	Having a medical condition that prevents implementation of study procedures
Living in a house that consented to be enrolled in the TIRS study	Temporary visitor to household
	Plans to leave study area within next 12 months Consent and assent not obtained

Engaging communities early in the trial will be essential for maximizing participant acceptance and retention [46, 47]. An experienced team of 10 social workers, who will interact directly with study participants (through informal conversations, games, and other educational activities with children), will ensure they remain engaged throughout the duration of the study [47]. Several factors may lead one household to withdraw from the intervention. Householders may sell their home and move to a different location, and we will consider them lost to follow-up. Householders may refuse to receive the intervention on a second or third opportunity, meaning they will not be subject to treatment (and therefore excluded from any future analysis). Our team will document voluntary withdrawals and communicate them as part of the trial reporting.

### Trial performance milestones

Table 5 shows our proposed milestones for the trial, following the SPIRIT checklist, and sections below provide information on each step (see Additional file 3). They can be divided into (a) trial planning, (b) TIRS evaluation, and (c) trial analysis and reporting. Trial design will be finished during the first year. Enrollment is expected to last up to 3 months, when all 4600 children will enter follow-up. Trial evaluation will occur for two transmission seasons, with the possibility of adding a third season should incidence of the primary endpoint be lower than assumed. Trial analysis will include a projection of TIRS impact, based on results from the CRCT, using our stochastic simulation model fitted to our study population.

**Table 5:** Schedule of enrollment, interventions, and assessments (SPIRIT figure)

	Prior to start of clinical study	Enrollment	Within 12 months of enrollment	Within 12–24 months of enrollment	Within 24–36 months of enrollment	Within 36–48 months of enrollment (possible 3rd season)
**Enrollment (year 1)**						
**Cluster eligibility screen**	X					
**Individual eligibility screen**	X					
**Informed consent**		X				
**Allocation**			X			
Interventions* (years 2–3)						
***TIRS***_***1***_				X		
***TIRS***_***2***_					X	
***TIRS***_***3***_						X
***No activities in untreated clusters***				X	X	X
**Assessments**						
***Baseline: entomology, seroprevalence***			X			
***Active surveillance/entomology***				X	X	X
***Annual sero-survey***				X	X	X
***Mobility surveys***			X	X	X	X
***Monitoring insecticide adverse effects***				X	X	X
***Laboratory testing***			X	X	X	X

*Routine vector control in response to symptomatic ABV disease will not be discontinued

#### Baseline study

A baseline assessment of household characteristics (size, building materials, number of rooms, number of inhabitants) and *Ae. aegypti* infestation and susceptibility to insecticides will occur July–December 2020 (Fig. 2). Entomological collections will be conducted monthly in 10% of all houses located in the centers of the clusters (blue blocks in Fig. 2, equal to 1350 houses across 50 clusters). Standard ovitraps will be placed to collect eggs that will be reared for assays to characterize insecticide susceptibility in mosquito populations. After the transmission season (January–April 2021) and during individual child enrollment, a baseline sero-survey will quantify levels of ABV seroprevalence. All enrolled children will provide a blood sample by venipuncture, which will be tested for the presence of neutralizing antibodies against DENV, CHIKV, or ZIKV (see laboratory methods below).

#### Intervention

Personnel from the Servicios de Salud de Yucatan (SSY; Yucatan’s Ministry of Health) will conduct the TIRS after proper training [48]. Based on our model predictions [32], spraying should start May–June and extend for 1–2 months. For 25 treated clusters with a total of approximately 625 city blocks and an average of ~ 30 houses per city block, we calculated a total of 18,750 houses to be treated. Assuming an average spraying time of 15 min per house, we estimated a workforce of 24 spray technicians is needed. To date, *Ae. aegypti* in Merida are susceptible to insecticides from both the carbamate and organophosphate classes [49]. We will prioritize the use of the organophosphate pirimiphos-methyl (Actellic 300CS®), given its longer residual power in comparison to the carbamate bendiocarb (Ficam®) [50]. However, if insecticide resistance profiles of mosquitoes after the first year of spraying show decreases in susceptibility to the active ingredient in Actellic 300CS®, we will switch to Ficam®. Insecticide application will follow strict procedures developed by project team [48]. Residents will be asked to temporarily leave the house during treatment and wait 1 h for the product to dry before re-entering. Staff will wear branded uniforms with identification and use appropriate personal protective equipment.

#### Intervention evaluation

**Fig. 3:**
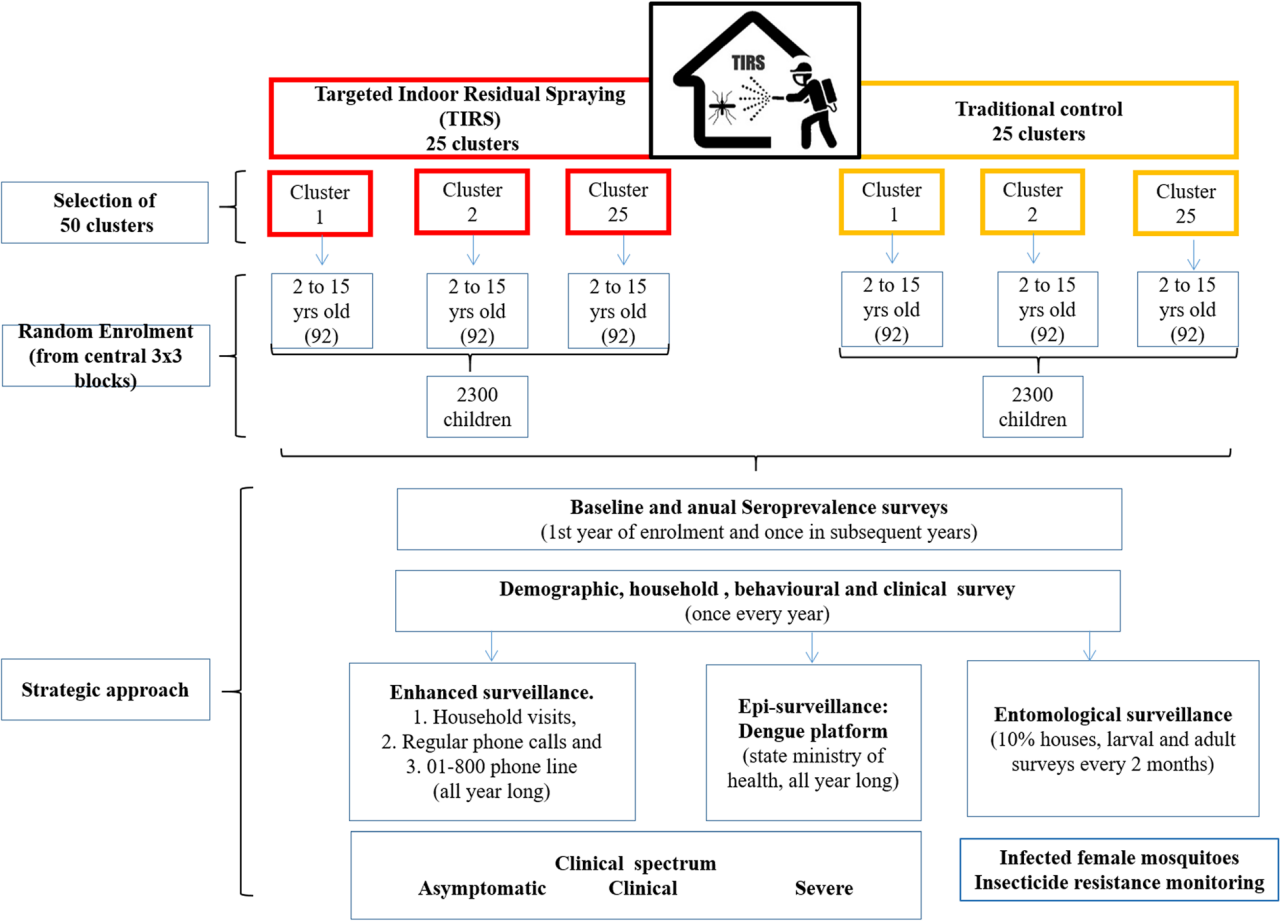
Proposed trial components

The epidemiological impact of TIRS on the primary endpoint will be evaluated by active surveillance to detect and lab-confirm symptomatic DENV, CHIKV, or ZIKV from July 1 to December 31 of each season (Fig. 2). Enhanced symptomatic ABV case detection will rely on three sources (Fig. 3). Ten field teams consisting of a nurse and a social scientist will conduct wellness visits to all enrolled children once per week, with the goal of identifying any probable case of ABV illness. In addition to wellness visits, nurses will call parents/guardians of enrolled children regularly (twice per week) to check for the occurrence of any ABV symptoms. When interacting with parents/guardians, nurses will also remind them that they can call our toll-free 01-800 number in case of any illness compatible with an ABV infection. Widely used by the previous cohort, the 01-800 number enhanced the detection of symptomatic individuals by providing study participants 24-7 access to a toll-free phone number to consult an “on call” project physician about any symptom in their children [37]. Additionally, our project will access the online ABV database managed by Mexico’s National Center of Preventive Programs and Diseases Control (CENAPRECE) [51] to identify all reported symptomatic cases (including all ages, not only children) residing within study clusters in real time, and to map routine vector control actions performed by SSY.

For ascertaining the primary endpoint, a suspected symptomatic ABV case is defined as a participant with acute onset of fever (axillary temperature ≥ 38 °C) or a non-focal rash plus any additional symptom such as headache, conjunctivitis, arthralgia, or myalgia. When a suspected ABV case is identified through active surveillance, they will be visited preferably on the same day by one project physician to perform a physical examination (physical exam, temperature, vital signs). The doctor will be joined by one field team member, who will obtain demographic and behavioral data, and collect blood specimens. Acute and convalescent (obtained 28 [range 21–35] days after symptom onset) blood specimens will be collected from each suspected case to confirm ABV infection. Additionally, history of movement (by a retrospective movement survey) [16, 52] will provide information on potential exposure locations for each case. After laboratory confirmation, participants will meet with study physicians, who will explain the diagnosis and potential steps if symptoms worsen.

Epidemiological impact will be further assessed via a secondary endpoint capturing serological evidence of ABV infection (Table 1). Yearly blood samples from all enrolled participants will be collected after the regular transmission season (from January to April) to test for serologic evidence of interval infection by DENV, CHIKV, or ZIKV, as in [37–39]. In addition to collecting blood specimens, project team will also conduct annual prospective movement surveys to characterize the routine mobility patterns of participants.

Entomological impact will be measured by standardized monthly collections of indoor adult *Ae. aegypti* (Table 1). A random sample of 10% of the houses located in the center (“yolk” of our fried egg design) of each cluster (~ 1350 houses in total) will be visited and surveyed for the presence of adult *Ae. aegypti* mosquitoes indoors using Prokopack® aspirator collections performed for 10 min per house, as described in [31, 53]. Female *Ae. aegypti* collected indoors will be pooled by city block and tested for ABV infection. Entomological surveys will begin immediately following TIRS implementation (July 1) and will be performed monthly for 6 months (until Dec 31). Monthly WHO cone bioassays [31, 54] will be done in a random sample of 25 treated houses to monitor the residual efficacy of the insecticide used.

### Collection and storage of specimens

Venipuncture procedures will be performed using standard aseptic techniques. An experienced phlebotomist will take the blood sample from an antecubital vein. Blood will be collected into Vacutainer® collection tubes or by a needle and syringe. A 22-gauge needle will be used for 5–15-year-olds, and a 23-gauge needle for children < 5 years. Blood specimens will be immediately taken to Yucatan State Diagnostics laboratory, dependent of the Ministry of Health for immediate molecular diagnostics (acute samples) or serum separation, followed by ELISA tests (convalescent samples and annual blood draws). Aliquots of all specimens will be stored at − 70 °C in labeled polypropylene cryogenic vials at UADY, and then transported to Emory University for advanced diagnostics. Long-term specimen storage will occur at Emory University. Specimens from individuals who did not sign the “future use” clause of the consent will be discarded after diagnostics, following sample processing procedures established by Yucatan State laboratory.

### Laboratory plan

**Fig. 4:**
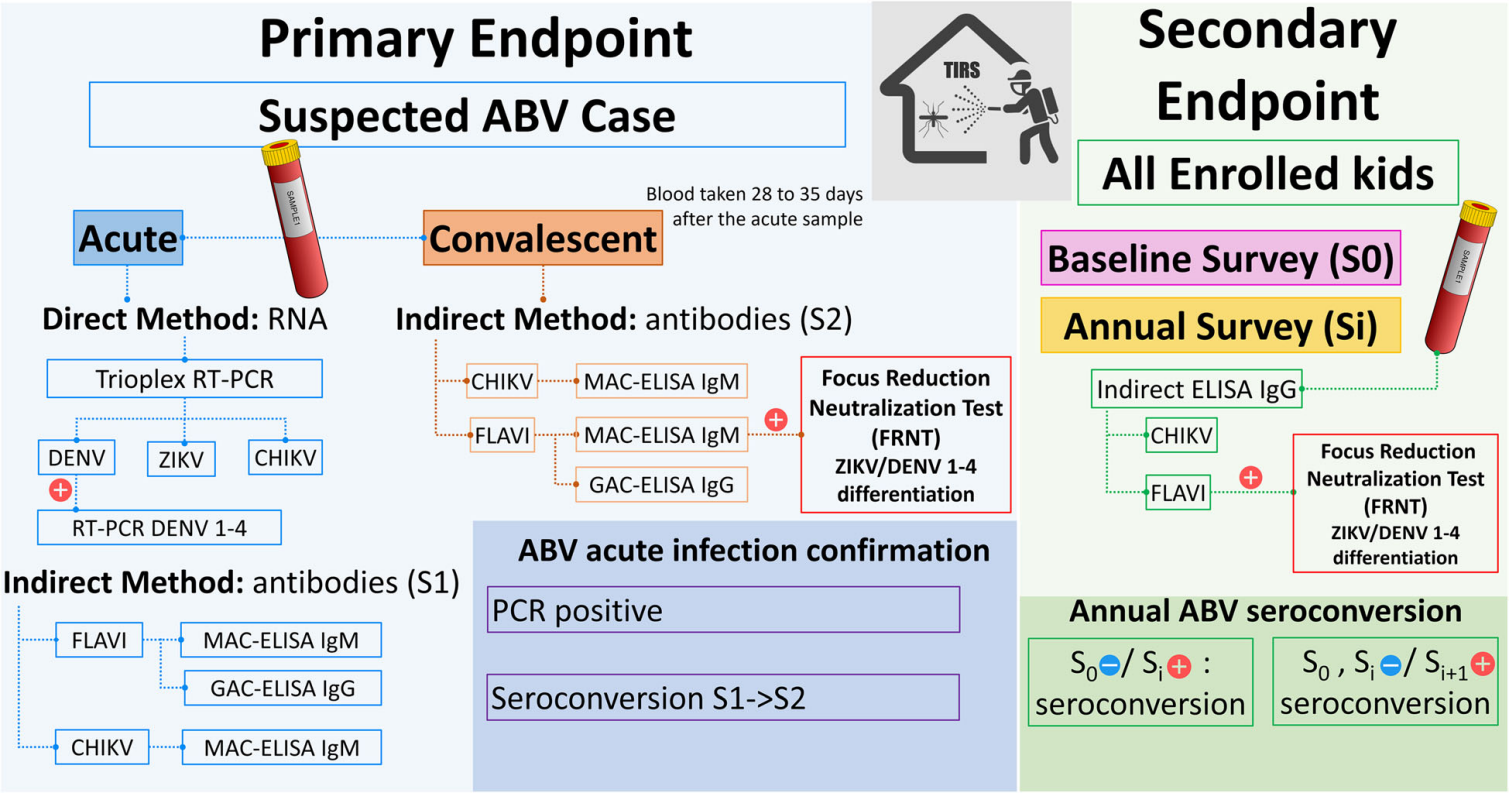
Algorithms for laboratory confirmation of acute ABV infection and annual seroconversion. Divided by phase of specimen collection, active surveillance of acute infections (right) or annual serological study (left)

Figure 4 shows all lab testing components of the trial, which will occur at SSY, UADY and Emory University. Acute samples from active surveillance will be tested at the Yucatan State Laboratory using a multiplex reverse transcriptase-polymerase chain reaction (RT-PCR) [55] and virus-specific IgM ELISAs. Annual serologic samples will be tested at Yucatan State Laboratory by antigen capture ELISA for human IgG [56], and positive samples will be taken to Emory University for focus reduction neutralization testing (FRNT) [57–59]. Natural ABV infection rates in *Ae. aegypti* will be detected by RT-PCR [55] at UADY. Standard CDC bottle bioassays [60] will assess phenotypic resistance of adult *Ae. aegypti* from treatment and control clusters pre-intervention and at 3 and 9 months post-intervention every year. F0, or F1 progeny, from field-collected eggs will be screened for susceptibility to pirimiphos-methyl [60]. If resistance is detected, both DNA and RNA will be analyzed from a subset of the phenotyped mosquitoes to calculate the frequencies of known resistance alleles as well as expression of resistance-associated genes.

### Case diagnosis

Given cross-reactivity and variable sensitivity of assay methods, we will use a composite approach to diagnose ABV infections (Fig. 4). For active surveillance, two diagnoses are used: preliminary diagnosis—suspected cases are confirmed if RT-PCR is positive for any ABV. If RT-PCR is negative, the acute specimen IgM result is considered and any positive IgM result indicates a preliminary diagnosis of ABV infection. If both ZIKV and DENV IgM assays are positive, the case is designated as a case of flavivirus infection. Final diagnosis—paired acute and convalescent specimens will be tested for IgM and IgG seroconversion. These results will refine the case designation. A case with laboratory evidence of ABV infection in the acute testing must also demonstrate seroconversion or increasing levels of IgG or IgM in the convalescent specimen. RT-PCR+ suspected cases that do not exhibit seroconversion or increase in IgG or IgM levels will be designated recent infections, but not ABV cases (that is, this result is most consistent with an etiology other than ABV infection as the cause of the symptomatic illness). Additionally, IgG or IgM seroconversion or increasing IgG that is observed when RT-PCR and acute specimen IgM are negative will be considered confirmation of an ABV case. This approach may increase the sensitivity to detect ABV cases that have false negative PCR testing and have not yet mounted an IgM response at the time of presentation. Finally, if convalescent serology does not distinguish between DENV and ZIKV infection, the annual surveillance sample for that subject will be considered. If it is clear from neutralization testing on the annual surveillance specimen what the intervening viral infection was, that will become the designation of the ABV case captured during active surveillance.

The annual serologic surveillance takes into account that the majority of ABV infections are inapparent. It will also account for the known cross-reactivity among ABVs. CHIKV is an alphavirus, and serologic assays for CHIKV perform with high sensitivity and specificity. ELISA is likely sufficient for annual CHIKV serosurveillance. DENV and ZIKV are related flaviviruses, and conventional approaches to serologic diagnosis of flavivirus cases can exhibit reduced specificity. However, the antibody response to DENV and ZIKV is dynamic, and cross-reactive antibody levels are greatest in the first few months after infection. Thus, cross-reactivity is present but less intense in late convalescence, which is one reason for performing serosurveillance in the low-transmission season. For flavivirus surveillance, neutralizing antibody titers will be compared using the FRNT50 (inverse of serum dilution that exhibits 50% of maximum neutralization). Conversion of neutralization assays from negative to positive in subsequent years is strongly supportive of interval infection. The precise infecting virus (DENV1–4 serotype or ZIKV) can often be identified by comparing relative FRNT50 values for each virus. A ≥ 4-fold difference in the FRNT50 is considered a significant difference. Once an individual has high titers to multiple DENV serotypes, detection of additional DENV infection is challenging by serosurveillance alone. The details of interpreting all possible flavivirus neutralizing antibody profiles are beyond the scope of the article. We have reviewed the key concepts recently [61].

### Statistical considerations

#### Primary analysis

The primary analysis will estimate the overall efficacy of TIRS in reducing the rate of laboratory-confirmed ABV illness, where the overall efficacy is estimated as one minus the hazard ratio from a Cox proportional hazards model [62]. The hypothesis test for the primary outcome will be a score test of the null hypothesis that TIRS efficacy is 0; the two-sided test will be conducted at the *α* = 0.05 level.

The Cox proportional hazards model will be fit using individual-level data for eligible and consenting children. The primary endpoint will be time to symptom onset of first laboratory-confirmed ABD. The time origin will be July 1 prior to the first season, by which time spraying will have been completed. The analysis will consider events occurring between July 1 and December 31 of each year of the study, as this corresponds to the time when the residual effect of the insecticides used in TIRS is expected to be active and while active surveillance is ongoing. To account for clustering, the model will include a robust variance estimator with two parameters; one characterizes the level of correlation in outcomes between children within the same household, and one characterizes the level of correlation in outcomes between children in different households but within the same cluster. We will use Schoenfeld residuals to assess departures from proportionality, as would occur if the effect of TIRS varies over time [63]. We will use time-dependent (piecewise) models where significant non-proportionality occurs [64].

#### Secondary analyses

Planned secondary analyses of clinical and human serological data include:

- Cox proportional hazards model with time to first laboratory-confirmed symptomatic ABV disease as the endpoint, adjusting for additional cluster- and household-level covariates (e.g., population density, household size, socio-economic status).
- Cox proportional hazards model with time to first laboratory-confirmed symptomatic ABV disease as the endpoint, adjusting for routine human movement as measured by the prospective movement survey (measured in all enrolled participants). The proportion of time in treated areas will be included as a further covariate, as described in [10].
- Disease-specific versions of the primary analysis (e.g., time to first laboratory-confirmed symptomatic dengue disease as the endpoint), if data permit.
- Analysis of recent human movement measured by a retrospective movement survey in enrolled participants presenting with symptoms for laboratory confirmation. The data will be analyzed using a test negative design-type structure, where individuals testing negative for any ABV will serve as a comparator group for individuals testing positive for ABV. The analysis will adopt recently developed methods for cluster randomized vector control trials [65, 66].
- Binomial generalized linear mixed effects model to assess the efficacy of TIRS for reducing laboratory-confirmed DENV, CHIKV, or ZIKV infection will be analyzed as cumulative incidence over the two (or potentially three) transmission seasons, as measured from annual serological samples. Given the larger number of sub-clinical and undetected ABV infections compared to symptomatic ABV illness, the study will be amply powered to detect a statistical difference in ABV infections (measured by annual serology).
- Using the passive surveillance data, we will quantify the community impact of TIRS on symptomatic ABV cases reported to the public health system, beyond our pediatric cohort. Poisson regression will be used to compare cluster-level incidence rates across trial arms.
- Acceptability of TIRS intervention will be assessed by calculating summary statistics from the post-intervention data. Acceptability measures will be paired with any adverse reactions experienced or reported by study participants and assessed by our team of physicians.

For mosquito data, planned secondary analyses include:

- The following *Ae. aegypti* adult indices will be calculated for each sampling date and compared between treatments and over time: presence (binomial variable) and abundance (count variable) of adults, females, and blood-fed females per house. Generalized linear mixed effects models (GLMM) nested at the cluster (level 1) and city block (level 2) levels will be used to compare each entomological index between treatment and control arms, as in [31]. Link functions for GLMMs will be binomial for presence indices and negative binomial for abundance indices. The best fit models (after comparing AIC values for models including all levels or only level 1) will be used to calculate odds ratios (OR; for mosquito presence/absence) and incidence rate ratios (IRR; for mosquito abundance) using control houses as the unit of comparison. We will calculate the operational efficacy of the intervention as *E* = (1 − IRR) × 100. This measure, ranging between 0 and 100, describes the percent reduction of mosquito abundance in treated houses with respect to the control.
- Similarly, a negative binomial GLMM will test for differences in treatment and control arms for infection rates with DENV, CHIKV, or ZIKV, calculated as minimum infection rate, following similar statistical methods as for *Ae. aegypti* abundance.

##### Entomological correlates of ABV transmission

- Epidemiological and entomological information will be combined to quantify the relative reduction in the incidence of symptomatic ABV illness at the cluster level observed from a measured entomological reduction due to TIRS (measured as number of adult or female *Ae. aegypti*). Binomial GLMMs, with random intercepts at the cluster and year levels, will quantify the association between both variables for the duration of the trial and provide values of threshold vector densities associated with a significant reduction in the odds of human symptomatic infection.

##### Transmission modeling

Our existing mathematical model for Yucatan [32, 67, 68] will simulate the effectiveness of TIRS for different scenarios of intervention coverage and insecticide residual power, using the observed trial data as a critical model input. This agent-based model of individual people and mosquitoes incorporates household demography, a spatially heterogeneous population structure based on census and remote sensing data, movement of workers and students, and seasonal fluctuations in mosquito population and incubation period. Different movement (e.g., mosquito vs. human) and transmission (e.g., pathogen introduction and elimination) dynamics become relevant at different spatial scales; thus, we will predict the impact of scaling up TIRS to the entire state rather than treating just Merida. Simulating epidemiological trends of scaled-up TIRS for periods longer than the duration of this trial (e.g., a decade) will evaluate the effect of changing population-level immunity and generate measures of effectiveness that are more informative for programmatic decision making.

### Data management

**Fig. 5:**
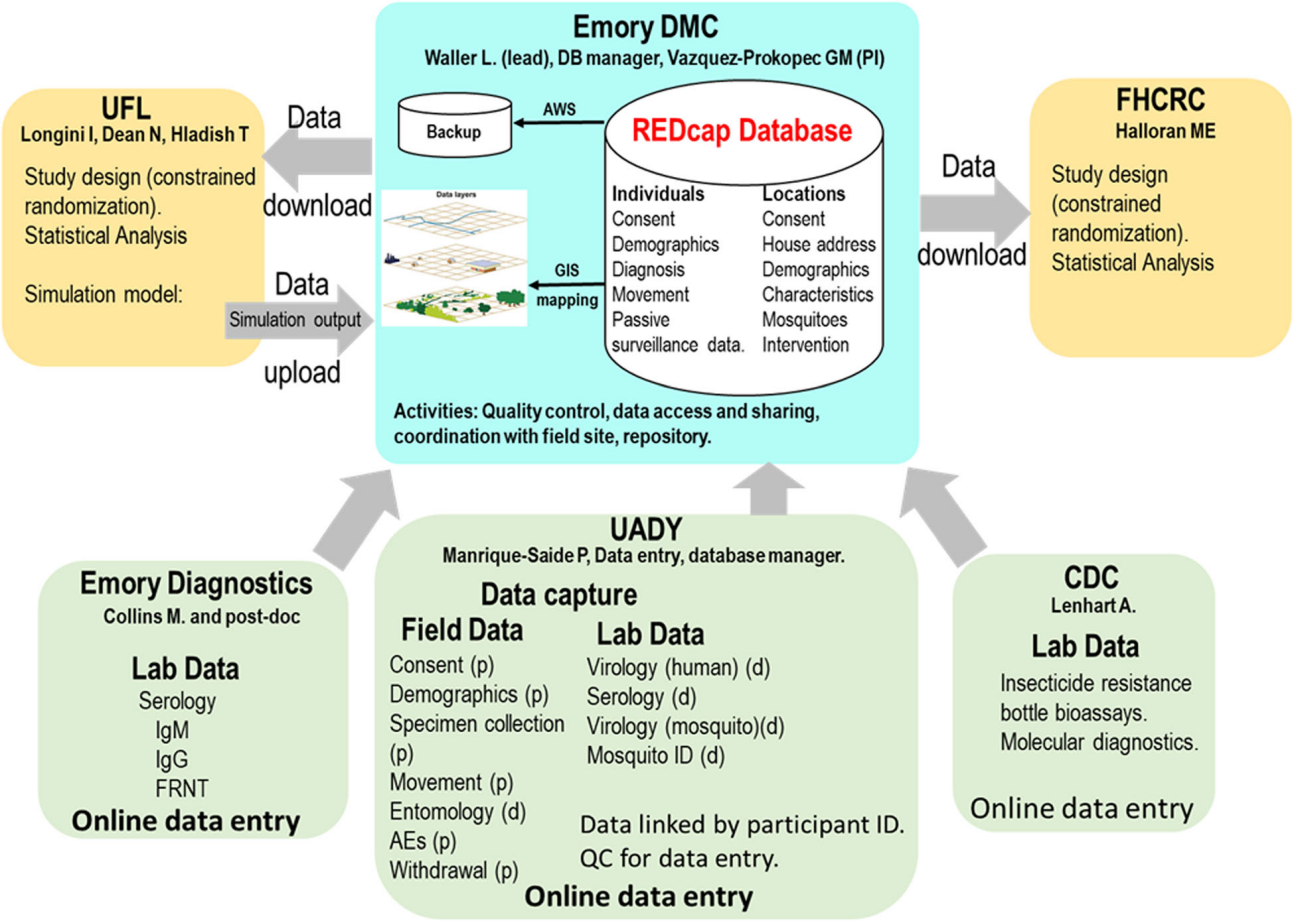
Structure and organization of the Emory data management core (DMC)

Emory University will coordinate all aspects related to data storage, management, and sharing. A data management core (DMC) provides timely and efficient curation and dissemination of study data from multiple sources (e.g., clinical, laboratory, passive surveillance, entomology, demographic, Ministry of Health interventions), all essential to the success of the trial (Fig. 5). Information from the trial including consent forms, surveys, active surveillance forms, laboratory diagnostics, entomological surveys, mobility surveys, withdrawal forms, intervention acceptability, and annual blood draws will be collected in paper form and digitally recorded into our REDcap database (see below) by the data entry staff at UADY. Staff will enter information in a private dedicated space at UADY-UCBE. Laboratory results at Emory University will be entered directly into the REDcap database by laboratory staff using an online form. All forms were developed by our team specifically for this study.

All data will be stored on secure data servers and kept strictly confidential (with participant identifiers blinded by using non-identifiable IDs). Households are assigned codes unique to the project database, which are then used to identify all subsequent data we will collect. Outside of the database, these codes will not be interpretable, rendering the data effectively unidentifiable without access to our servers. Blinding of identifiable data will occur in the analysis stage also. All diagnostics of specimens will be conducted using the sample ID, blinding laboratory personnel from any identifiable information or membership of samples to a given study arm.

Access to the database will be primarily administered through a custom, web-based interface with restricted access privileges and encrypted data transfer (REDcap, https://www.project-redcap.org/). Different data entry interfaces will be generated for each component. Access will be limited to certified project personnel and certified associates, who will be provided unique login and password combinations. Database servers will be protected by multiple layers of security. Databases will be shared electronically through secure servers among key project personnel for analyses, publications, oral presentations, and project development. Regular checks of the database for completeness and accuracy will be performed.

### Trial continuation rules

The heterogeneous nature of ABV transmission may dictate the need for a third transmission season to evaluate the epidemiological impact of TIRS. The decision to continue into a third season will follow an event-driven decision process. After the second season evaluating TIRS, the statistical team will quantify the number of total primary endpoints. We will pursue the following ranking in order to evaluate whether to stop or continue into a third season:

- If 90+ primary endpoints are detected, stop and analyze data as final.
- If 20–89 primary endpoints, continue into a third season.
- if < 20 primary endpoints, examine feasibility/futility.

The choice of 90+ endpoints is based on our power calculations and represents the target number of events expected for a power of 80% and a TIRS efficacy of 70%. The choice of < 20 endpoints represents the target number of events needed for a power of 80% when TIRS efficacy is 90%.

### Monitoring adverse effects (AE)

Overall, the risks to study participants are minimal in all of our study procedures (Table 6). The most serious risk is related to potential intoxication with the insecticides used in TIRS (Table 6). Both Actellic 300CS® and Ficam® have been approved by the World Health Organization (WHO) for indoor control of mosquitoes [18, 19]. The WHO’s hazard assessments concluded that, when used for indoor residual spraying as instructed and at the recommended doses, both products do not pose undue hazards to the spray operators or residents of the treated dwellings [69–71]. Provided that operational guidelines are followed, routine cholinesterase monitoring of spraying personnel during indoor residual spraying programs is not required [69–71].

**Table 6:** Potential risks associated with specific components of our study

Study component	Risks
Intoxication due to unintended exposure to insecticides	Direct (contact) or indirect (inhalation of fumes) intoxications are rare but likely. Bendiocarb: Symptoms of poisoning include excessive sweating, headache, chest tightness, giddiness, nausea, vomiting, stomach pains, salivation, blurred vision, slurred speech, and muscle twitching.
	Pirimiphos-methyl: can cause cholinesterase inhibition in humans; that is, it can overstimulate the nervous system causing nausea, dizziness, and confusion.
Febrile surveillance Longitudinal cohort	Pain or discomfort, bruising, or infection at venipuncture site or temporary dizziness during blood draw Use of identifiable information (demographic information, address, febrile status)
DENV+, CHIKV+, and ZIKV+ participants	Same as for febrile surveillance and longitudinal cohort The data gathered in this project will be identifiable and certain data types, such as movement interview, are sensitive. The primary risks lie with identifying the individuals who provided information they consider confidential (e.g., movement to private locations). There is a small risk that the repeated blood collections will cause or exacerbate anemia.
In-depth interviews (prospective and retrospective movement interviews)	Risks to study participants are minimal. Participants may feel that in-depth interviews take up too much time—but they have the option of ending their participation at any time. There are no sensitive topics covered, but if any participant feels that there is something he/she does not want to talk about, he/she does not need to answer all questions.

#### Evaluation of AEs

During the period of active surveillance, immediately after TIRS application, study participants will be contacted regularly (1×/week in-house or 2×/week by phone calls) by our team, who will ask for the presence of any sign of intoxication in any of the members of the house. Such contacts will coincide with the epidemiological evaluation of the intervention. In addition to our team’s direct contact, households receiving TIRS will receive a pamphlet with a 0–1800 toll-free number for them to self-report any signs of intoxication. Once in the presence of a probable case of intoxication, a physician will medically assess the patients to diagnose the extent of their condition. Vital signs, together with respiratory distress (i.e., bronchorrhea, bronchospasm) and clinical evidence of cholinergic excess (i.e., salivation, vomiting, urination, defecation, miosis), will be followed until they resume. In cases of severe intoxication, plasma cholinesterase activities will be assessed, together with electrolytes and serum lipase (both tests can be performed at UADY’s School of Medicine Public Health Laboratory, which routinely performs such tests for pesticide occupational exposure assessments). Given the insecticide dose and mode of application used in TIRS, we expect most intoxications to be mild and resume after exposure ends (i.e., after individuals are exit their home). Our preliminary results from our Phase II entomological trial utilizing Actellic 300CS showed that in 160 houses (including 630 individuals) a total of 19 cases (3%) of symptoms compatible with a reaction to the insecticide were detected (Vazquez-Prokopec et al. unpublished). The most common signs (accounting for 85% of symptoms) were headache, nausea, and mild skin irritation. However, if the physician considers that a moderate to severe intoxication occurred, serological tests will be performed to confirm the cause of their condition.

#### AE reporting

All probable AEs will be noted in the adverse event log (AEL), which will be the primary form of communication between physicians and the PI. AELs will be filed immediately (one record per event) after the detection of a probable AE (the form will include links to any specific medical record or laboratory record associated with each case). Once an AEL is filed in the database, the PI will receive an alert requiring his attention. Upon conversation with the study doctors, the PI will make an informed decision as to whether the condition represents a reportable AE or not. Any AE or unanticipated problems (UP; serious, life threatening, or result in death and unexpected and caused by the intervention) involving risk to participants will be notified to the IRB within 10 calendar days of their occurrence. Emory IRB will generate specific forms within their eIRB platform to report any AEs or UPs associated with this study. The IRB reports on AEs or UPs will be received by the NIH program officer assigned to this study. In the unlikely situation that UPs emerged due to TIRS implementation, Emory IRB and the NIH program officer will coordinate with the PI about the temporary or permanent suspension of this study.

### Trial organization

This project will strengthen a unique US-Mexico partnership involving universities and research centers (Emory, UADY, Fred Hutch, UF) and federal agencies (CENAPRECE, Mexico’s National Institute of Public Health, CDC) together with state agencies (SSY). Emory University will lead the project and will be in charge of overall coordination, procurement of commodities (e.g., insecticides, diagnostic reagents), and data coordination, advanced diagnostics, and IRB approval. The Autonomous University of Yucatan will coordinate all aspects of the field implementation of the trial as well as the integration of field and laboratory data streams. Trial design will be led by Fred Hutchinson Cancer Research Center. Analyses for the primary and secondary endpoints as well as for evaluation of trial continuation will be conducted by UF (Ira Longini, Natalie Dean), with input from biostatisticians from Fred Hutchinson Cancer Research Center. UF will also lead the mathematical modeling component. Technical support will be provided by the US CDC to evaluate patterns of insecticide resistance in space and time. Mexico’s CENAPRECE will provide access to the online ABV database. The SSY will contribute spraying personnel and access to samples for laboratory testing in support of the trial’s active surveillance procedures, as well as help with communication about TIRS and the trial’s goals. Dr. Silvina Contreras-Capetillo, MD (Hospital O’horan, Merida, Mexico), expert in clinical aspects of *Aedes* viruses, particularly genetic malformations in Zika, will act as an independent trial monitor. The funder (NIH) considered the low risks associated with the intervention not to merit the establishment of a DSMB. As such, the study team and the NIH program officer(s) will communicate directly about study findings, reports from independent trial monitor, continuation rules, and adverse events. Any deviation from protocol will require prior approval by the NIH program officer.

### Ethical considerations

The study protocol and associated documents including informed consent forms are approved by the respective Institutional Review Boards (IRB) of all collaborating institutions as well the National Institutes of Health. The trial protocol was registered on clinicalTrials.gov (NCT04343521) on April 13, 2020. It will be made clear during the consent process that no information can be shared with anyone other than designated study personnel, the paper and computer files will be well protected, and we will ask that interviews be carried out one-on-one to prevent other family members listening in. Consent and assent forms include a separate section where participants give permission to the PI to keep their specimens for future tests or studies. We will take all necessary measures to ensure confidentiality. It will also be made clear to study personnel that any violation of confidentiality would be a fireable offense. All paper data forms will be stored in locked files or cabinets in UADY in a specified storage facility with limited access. Access to computer data files will be password protected to allow exclusive access to appropriate study personnel. The paper data forms associated with the project (e.g., consent forms, questionnaires, census) will be stored in accordance with IRB regulations. Should consent be given for future use, then serological samples will be stored indefinitely. The samples will not have any participant identifiers, beyond the participant’s code. If, however, consent for future use is not given, the blood samples will be destroyed immediately (using strict protocols at UADY for disposal of biological samples) following completion of the project. Monitor evaluations will occur once a year and will be timed to occur right after the epidemiological evaluation of TIRS (January–March). On every visit, Dr. Contreras-Capetillo will file a Monitoring Log and a Self-Monitoring Tool form. Self-monitoring will be performed on a random selection of 10% of study participants. The monitor will also review records of all adverse events as well as the information of any dropouts that occurred between monitoring periods. After the visit, the monitor will submit the Self-Monitoring Tool to the PI, together with any recommendations based on the visit. A phone call between the monitor and the PI will be scheduled, should corrective actions be required.

## Discussion

Novel tools and strategies that are operationally feasible and widely scalable are desperately needed to prevent and control ABVs. This Phase III CRCT trial will quantify the epidemiological impact of TIRS in preventing ABVs and generate a definitive evidence base for assessing the public health value of this approach.

The heavy reliance on pyrethroid insecticides for mosquito control has led to widespread pyrethroid resistance on a global scale [72]. The high levels of resistance to pyrethroids found in Mexico [73], including the Yucatan [49], prompted CENAPRECE to expand the chemical groups used for *Aedes* control to other insecticide classes such as carbamates and organophosphates, to which local *Ae. aegypti* are susceptible [49, 73]. A recent entomological CRCT performed in Merida, Yucatan, demonstrated that utilizing an insecticide to which *Ae. aegypti* were susceptible had a significant impact on indoor mosquito density, as compared to the use of a pyrethroid to which the local population was resistant [31]. The selection of new insecticide formulations (e.g., micro-encapsulated insecticides) with longer residual power (ca. 5–7 months) can further increase the effectiveness of TIRS. Fortunately, R&D for new insecticide formulations as well as novel chemistries for vector control has expanded, and new products are at various stages in product development pipelines [74]. Findings from this trial will not only aid in understanding how residual insecticides can function effectively for ABV control but also help catalyze R&D for residual insecticide formulations better suited for the surfaces and materials found in urban areas.

Responding only to symptomatic ABV cases likely misses a significant number of cases as a large proportion of ABV infections are asymptomatic, which can still successfully infect mosquitoes [75] and in turn significantly contribute to ABV transmission [17]. Findings from a spatially explicit agent-based model of dengue dynamics in Yucatan, Mexico [32, 67, 68], suggested that TIRS maximal effectiveness occurs when it is deployed preemptively (before the seasonal peak of ABV transmission) rather than reactively. Our trial will evaluate the preemptive implementation of TIRS (spraying 1–2 months prior to the beginning of the peak ABV transmission season). If found efficacious, the trial will make a strong case for the public health value of preemptive, long-lasting vector control measures against ABVs. This finding would contribute to a paradigm shift in *Aedes* control and ABV prevention, leading to innovations in the way that interventions are conceptualized and brought to scale in operational settings.

While the CRCT approach itself is largely standard, focusing on adherence to core epidemiological principles [76], our trial will incorporate several innovative features into the randomization and analysis. We have modified the covariate-constrained randomization procedure [44] to include a selection step to maximize the geographical spread of the clusters. This strategy may be useful in future vector control trials. Through the use of highly spatially resolved prospective and retrospective movement surveys, we will be able to refine our estimates of TIRS efficacy to account for participant time spent in treated and untreated areas [10]. Finally, we are able to directly integrate trial data on mosquito abundance, human movement, and clinical outcomes into an existing mathematical model to better understand the potential population-level impacts of TIRS. Using statistical simulations to help interpret and contextualize the results of an infectious disease trial is an emerging area of research [77]. To fulfill the critical need for carefully designed trials for vector control [15], this study will provide key data on the epidemiological impact of TIRS on ABVs and contribute methodologies and approaches for the design of future CRCTs.

## Trial status

At the time of submission, the project is on its second trimester (Table 5) and main administrative activities have been activated. Initial community contacts are expected to occur on mid-October 2020, with concurrent participant enrollment (level 2) and baseline serology occurring January–March 2021. Such timeline differs 3 months from the original proposed plan, due to the COVID-19 contingency that has limited presence of field personnel accessing households. Protocol version 2.0: July 14, 2019 (approved on August 1, 2019, by NIH/NIAID/DMID and on November 12, 2019, by Emory University IRB).

## Supplementary information

**Additional file 1.** WHO Trial Registration Data Set (Version 1.3.1): checklist.

**Additional file 2.**

**Additional file 3.** SPIRIT 2013 Checklist: Recommended items to address in a clinical trial protocol and related documents.

## Data Availability

The full trial protocol will be made publicly available within 1 year of the conclusion of data collection. The datasets generated in this study will be made available by the corresponding author on reasonable request, within 1 year of the conclusion of data collection.
