# Supplementary material for: The TIRS trial: protocol for a cluster randomized controlled trial assessing the efficacy of preventive targeted indoor residual spraying to reduce Aedes-borne viral illnesses in Merida, Mexico
Source: Trials. 2020 Oct 8;21:839. doi: 10.1186/s13063-020-04780-7 (PMC7542575; doi:10.1186/s13063-020-04780-7)
Supplement: Supplementary file 1 — Additional file 1. WHO Trial Registration Data Set (Version 1.3.1): checklist. [file 13063_2020_4780_MOESM1_ESM.docx]

**WHO Trial Registration Data Set (Version 1.3.1): checklist**

*(number next to each section indicates page in protocol)*

[*https://www.who.int/ictrp/network/trds/en/*](https://www.who.int/ictrp/network/trds/en/)

| **Component** | **Present in protocol (page#)** | **If absent, description** |
| --- | --- | --- |
| 1. **Primary Registry and Trial Identifying Number** | **3** |  |
| 1. **Date of Registration in Primary Registry** | **3** |  |
| 1. **Secondary Identifying Numbers** | **30** |  |
| 1. **Source(s) of Monetary or Material Support: Primary Sponsor** | **1, 30** |  |
| 1. **Contact for Public Queries** | **1** |  |
| 1. **Contact for Scientific Queries** | **1** |  |
| 1. **Public Title** | **1** |  |
| 1. **Scientific Title** | **1** |  |
| 1. **Countries of Recruitment** | **7** |  |
| 1. **Health Condition(s) or Problem(s) Studied** | **4-7** |  |
| 1. **Intervention(s)** | **8** |  |
| - 1. Intervention Description: | **12** |  |
| 1. **Key Inclusion and Exclusion Criteria** | **40** |  |
| 1. **Study Type**: |  |  |
| - 1. Type of study | **8** |  |
| - 1. Study design including: |  |  |
| - - 1. Method of allocation | **10** |  |
| - - 1. Masking | **NA** | No masking |
| - - 1. Assignment (single arm, parallel, crossover or factorial) | **9** |  |
| - 1. For randomized trials: the allocation concealment mechanism and sequence generation will be documented. | **10** |  |
| 1. **Date of First Enrollment** Anticipated or actual date of enrolment of the first participant. | **29** |  |
| 1. **Sample Size**: |  |  |
| - 1. Number of participants that the trial plans to enrol in total. | **9** |  |
| - 1. Number of participants that the trial has enrolled. | **29** |  |
| 1. **Recruitment Status** Recruitment status of this trial: |  |  |
| - 1. Pending: participants are not yet being recruited or enrolled at any site | **29** |  |
| 1. **Primary Outcome(s)** | **37** |  |
| 1. **Key Secondary Outcomes** | **37** |  |
| 1. **Ethics Review**: |  |  |
| - 1. Status (possible values: Not approved, Approved, Not Available) | **29** |  |
| - 1. Date of approval | **29** |  |
| - 1. Name and contact details of Ethics committee(s) | **29** |  |
| 1. **Completion date** Date of study completion: The date on which the final data for a clinical study were collected (commonly referred to as, "last subject, last visit"). | **24** |  |
| 1. **Summary Results**: |  |  |
| - 1. Date of posting of results summaries | **NA** | Not yet available. |
| - 1. Date of the first journal publication of results | **NA** | Not planned. |
| - 1. URL hyperlink(s) related to results and publications | **NA** | See item (h) |
| - 1. Baseline Characteristics: | **13** |  |
| - 1. Participant flow: | **45** |  |
| - 1. Adverse events: | **25** |  |
| - 1. Outcome measures: | **19** |  |
| - 1. URL link to protocol file(s) with version and date | **NA** | www.prokopeclab.org/TIRS (site going live Oct 2020) |
| - 1. Brief summary |  |  |
| 1. **IPD sharing statement** |  |  |
| - 1. Plan to share IPD (Yes, No) | **23** |  |
| - 1. Plan description | **23** |  |
