# Supplementary material for: The TIRS trial: protocol for a cluster randomized controlled trial assessing the efficacy of preventive targeted indoor residual spraying to reduce Aedes-borne viral illnesses in Merida, Mexico
Source: Trials. 2020 Oct 8;21:839. doi: 10.1186/s13063-020-04780-7 (PMC7542575; doi:10.1186/s13063-020-04780-7)
Supplement: Supplementary file 2 — Additional file 2. [file 13063_2020_4780_MOESM2_ESM.pdf]

INDIVIDUAL CONSENT FORM: EPIDEMIOLOGICAL MONITORING

Quantifying the Epidemiological Impact of Targeted Indoor Residual Spraying on *Aedes*-Borne Diseases.

Information for Parents

The Research Center "Hideyo Noguchi" invites your child to participate in a research study about the epidemiology of diseases transmitted by the mosquito *Aedes aegypti* (Dengue, Chikungunya and Zika), and the control of these vectors within Merida, Yucatan using insecticides sprayed inside the houses. This document explains why this study is happening, what this study will do and to help you decide if you agree that your child should participate in it. If any part of this document is not clear or you have any questions, please do not hesitate to ask one of the members of the team (Last page includes phone and email information from study principal investigator).

What are the diseases caused by *Aedes aegypti*?

Dengue, Chikungunya and Zika viruses are transmitted through the bite of the *Aedes aegypti* mosquito. People who become infected with any of these viruses may show different signs and symptoms.

DENGUE cases very often present fever accompanied by severe headache, vomiting, muscle and joint pains, pain behind the eyes and skin rash. Dengue fever evolves towards a more serious disease causing bleeding or a sudden decrease in blood pressure (shock). Dengue can be fatal in some cases, mainly in children. There are 4 serotypes of dengue virus (serotypes 1, 2, 3 and 4). Infection with one serotype protects against a second infection with the same serotype in the future; you may have an infection with the other 3 types and this increases the chance of having a more severe disease.

In the case of CHIKUNGUYA, there is significant pain in the joints and the sudden onset of high fever. Other symptoms may include headache, muscle pain, inflammation of joints or skin rash. The joint pain can often be intense and debilitating.

When a person has ZIKA, most times they are asymptomatic or they present rash, conjunctivitis (red eyes), headache, joint or muscle pains. The major risk is when a pregnant woman acquires the infection, as Zika can have some negative impact on the baby (congenital syndrome).

What is the purpose of this study?

We will evaluate the impact of indoor residual spraying (IRS) of insecticides in Merida, Yucatan, as an option for *Aedes aegypti* control. The impact will be measured by forming a cohort of children

2 to 15 years of age at the time of enrollment and follow them for three years to see if they acquire any *Aedes*-borne disease in areas sprayed with IRS and compared them to areas not sprayed.

How will this study develop?

This study will occur in the city of Merida, Yucatan, and last for **five years**. A cohort of 4,600 children will be enrolled on the first year, during October-January. Enrollment will occur through a home visit within the clusters identified by the team as being part of the study. During the first visit, a doctor from the team will verify if your son/daughter can participate in the study by performing a free medical examination of your child and asking you questions about his/her medical history. Only healthy kids with no serious health conditions will be able to participate.

During the duration of the study, your son/daughter will be visited once a year on a scheduled basis (between January-May) to provide a blood sample. Additional blood samples will be taken when your child presents fever (38°C or more) for at least two consecutive days, in order to check if fever is caused from a disease transmitted by *Aedes aegypti* or if it has another cause. This symptom-driven sample collection will occur during the typical dengue season, between July and December. Other procedures will involve short surveys to assess routine mobility patterns (and exposure to *Ae. aegypti*) and a survey to assess your satisfaction with the intervention and its impact.

Activity	Year 1	Year 2	Year 3	Year 4	Year 5
Annual blood draws (1/yr)	January-May	January-May	January-May	January-May	January-May
Wellness visits (1x week, 5min/visit)	None	July-December	July-December	July-December	None
Phone calls to assess health status (2x week, <5min call)	None	July-December	July-December	July-December	None
Symptom-driven blood sample collection (varies)	None	July-December	July-December	July-December	None
Healthy movement interviews (1yr, 20min visit)	January-May	January-May	January-May	January-May	January-May
Insecticide effect survey (1/yr, 15min visit)	None	July-December	July-December	July-December	July-December

For the symptom-driven sample collection, we will work with your family to make sure we can detect any episode of febrile illness in your child. The study staff will give you a thermometer so you can register any episode of fever (temperature greater than or equal to 38 C). During the dengue season (July – December), if your child has a fever of 38 C or more for at least two consecutive days, you will be prompted to contact the person in charge of the study via a 01800 0DENGUE hotline. Soon thereafter, the medical staff will arrange a home visit with the purpose of ruling out or confirming that your son/daughter has any of these *Aedes*-Borne Diseases. An initial blood sample of 10 mL would be collected within 5 days of the onset of fever to confirm diagnosis. If the project physician considers that your son/daughter courses has an *Aedes*-Borne Diseases, the project physician will ask some additional questions regarding your son/daughter and request a second blood sample two weeks after the first sample.

Contacts planned between visits

The staff participating in the study will contact you personally or by telephone calls and text messages, to monitor the health status of your child. Frequency of in-house visits will be 1x/week and the frequency of phone calls will be 2x/week.

What other procedures are planned?

The team consists of medical doctors and social scientist, whom you can contact or they will regularly contact you in order to keep track of any new information you wish to provide for the study.

What are the risks and undesirable effects to participate in the study and the blood sample?

Your son/daughter may feel a slight pain or a sting when the needle is inserted during a blood sample. He/she may also experience some throbbing on the site once the blood is drawn.

What happen will with their blood samples?

Blood samples will be sent to the Hematology laboratory of the Research Center Hideyo Noguchi UADY ***for immediate diagnosis***. Subsequently, the laboratory ***will*** safely store any unused portion of blood ***samples*** for at least five years ***after completion of the tests.***

Future use of blood specimens. If any part of their blood samples ***still remains*** after the tests, the researcher would like to keep it for ***their potential*** use in other investigations ***or research***. If you accept that your child will participate in this study, you will be asked if any parts of their blood samples that are not used, may be stored for other research purposes. If you accept, you will not be paid for allowing the use of these samples. If you do not agree, these samples will be used only for tests that are directly related to this study, including tests to confirm or rule out *Aedes*-Borne Diseases.

What will happen if you do not accept the participation or if you change your mind after agreeing to?

Participation in this study is completely voluntary. If you do not wish your child to participate, it is not required to grant their permission. In addition, if you agree to participate, but afterwards change your mind, you can withdraw at any time, without having to justify your decision. We will let you know of any new information generated during the study that might cause you to change opinion about the continuation of your child in the study.

The researcher –on the other hand- could remove your child from this study at any time; if there are any concerns about the health of your son/daughter or they are not following up the instructions of

the study. If your child leaves the study for any reason, the investigator might request to come back later to perform certain tests or examinations to check their health. All blood samples taken before retiring will be tested, unless you specifically request otherwise.

Are there any cost or financial compensation for participants?

Participants will not receive any payment for participating in this study. The participation of your son/daughter in this study is completely voluntary and will not have any additional cost for the children or their parents.

Which authorities are involved in this study?

The Research Center "Hideyo Noguchi" UADY will be the institution and headquarters of this study and is backed by the state health services and authorities. The Federal Ministry of Health (CENAPRECE) will also participate of this study.

Who will have access to personal and medical information of the participants?

The research information that we collect from your son/daughter during the study will be stored in protected and confidential databases; and only upon request can they be shared with other institutions and health authorities of Mexico. To protect your privacy, the medical histories of your son/daughter will be identified with a code and not by his/her name. Only the researcher and the staff involved in the study will know your name and other information that could link it to these records. You have the right to request and correct any error that may exist and to update the information collected form your child. Some information will be uploaded into ClinicalTrials.gov but in an un-identifiable format, meaning that neither your identity nor your children's identity will be included in the data.

If your son/daughter is hospitalized during the duration of the study, the researcher will need to access and verify the medical records. The results of the study may be presented in academic sessions (symposiums) or published in peer review journals. In any circumstance should your child data or identity will be reveal.

If you have any questions, please contact:

Dr. Norma Pavía Ruz, field team responsible RRC Hideyo Noguchi UADY
Telephone: 01800 0DENGUE, 9 245755.
Or the study's principal investigator:
Dr. Gonzalo Vazquez-Prokopec
gmvazqu@emory.edu

INDIVIDUAL CONSENT FORM: EPIDEMIOLOGICAL MONITORING

Study Title: Quantifying the Epidemiological Impact of Targeted Indoor Residual Spraying on *Aedes*-Borne Diseases

Principal Investigator: Gonzalo Vazquez Prokopec (Emory University)

This page to be filled out by research team at time assent is obtained:

Subject's Age: _____years (If the child is **younger than 6 years old,** assent is not required.)

Subject's Name: ________________________________

☐ **This child is 2 to 6 years old.**

____ ______________

Signature of Legally Authorized Representative **Date** **Time**

____

Printed name and relationship to subject of Legally Authorized Representative

---

Declaration of specific consent for the potential use of blood samples for research:

If you agree, indicate your choice by writing your initials in the following space:

____________ I authorize the use of my child's blood samples for future research by the investigator under the conditions described in this document.

MINOR ASSENT FORM: EPIDEMIOLOGICAL MONITORING

Quantifying the Epidemiological Impact of Targeted Indoor Residual Spraying on *Aedes*-Borne Diseases.

Information about this study:
Some mosquitoes may carry germs that can make people sick if those germs pass to them when bitten by a mosquito. Dengue, chikungunya and Zika are examples of sicknesses that come from mosquito bites.

Scientists and doctors often have ideas about how to stop people from getting sick, but they have to test those ideas in research studies. This study is testing a new way to kill mosquitoes inside of houses. For this new way, a worker sprays the inside of a house for about 10 minutes. That one time of spraying can kill mosquitoes for the next six months. We hope that doing this will mean that not very many people will get sick with dengue, chikungunya or Zika. But we need to monitor people to know if they get sick or not. You are being asked to be in this study because you live in an area where we are testing this new spraying method. If you agree to be in the study, you will be checked by a doctor to be sure you are healthy to participate. We will collect a blood sample from you at the start of the study and one time each year for three more years. Also, if you have a fever, we will check you to see if you might be sick with dengue, chikungunya or Zika. This will mean collecting a blood sample while you are sick and another blood sample two weeks later, once you are feeling better.

You do not have to be in the research study if you do not want to. Your doctors or your parents cannot make you be in the study if you do not want to. Even if you agree now, you may change your mind later. It is OK if you decide later to stop being in the study. You can talk to your doctors and parents or ask them questions to help you decide if you want to be in the study.

Study Title: Quantifying the Epidemiological Impact of Targeted Indoor Residual Spraying on *Aedes*-Borne Diseases

Principal Investigator: Gonzalo Vazquez Prokopec (Emory University)

This page to be filled out by research team at time assent is obtained:

Subject's Age: _____years (If the child is younger than 6 years old, assent is not required.)

Subject's Name: ______________________________

Check one box:

☐ **This child is 6 to 10 years old** — must obtain subject's verbal assent (subject's signature not required)

____

Signature of person soliciting assent of 6 to 10-year-old subject Date Time

---

☐ **This child is 11 to 17 years old** — subject's signature must be obtained for assent

____

☐ In my opinion, this child is unable to provide informed assent for non-age-related reasons, and the PI for this study has been informed of this determination.

- Reason(s):____

  ____

For subjects 11 to 17 years old: if you agree to be in this study, please sign your name below.

____

Signature of 11 to 17-year-old Subject Date Time

____

Signature of person soliciting assent of 11-17 year old Subject Date Time

Declaration of specific consent for the potential use of blood samples for research:

If you agree, indicate your choice by writing your initials in the following space:

____________ I authorize the use of my child's blood samples for future research by the investigator under the conditions described in this document.
